# Communication

**Published:** 1899-11

**Authors:** Leonard Pearson


### COMMUNICATION.

EDITOR JOURNAL OF COMPARATIVE MEDICINE, ETC.

*Sir:* I have recently received from Dr. James B. Rayner, of West Chester, Pa., the lower jaw of an ox that was killed in 1864.

This specimen shows enlargement opposite the first and second molars of the right side. The enlargement is about three inches long and increases the size of the jaw to twice its natural thickness. The outer surface of the enlargement is hard, but rough and porous, with numberless channels passing from the surface into the interior of the bone. The specimen has the appearance of a healed process. Dr. Rayner was called, in April, 1864, to see the animal from which this specimen was taken. He diagnosed actinomycosis (lumpy jaw), and considered it a regular typical case of that disease. Now, the interesting part of this history is that the steer was treated by administering on the feed a mixture containing iodide of potash, one-half drachm; iodine, three grains; and bone-meal, one tablespoonful, given three times a day. Externally, the enlargement was treated with an ointment containing iodine and iodide of potash. The condition improved during the summer, and by fall it was healed. The ox was killed for beef in the winter.

Attention was first called to the treatment of actinomycosis with iodide of potash by M. Thomassen, of Utrecht. Thomassen commenced to use this treatment in 1880, and it was upon his recommendation, and that of M. Nocard following it, that the treatment has been so extensively employed. It is now known that iodide of potash is a specific for actinomycosis.

How unfortunate it is that Dr. Rayner's case was not reported and published at the time. If it had been, others would have been prompted to have tried this treatment; its value would have been established more than thirty years ago, and Dr. Rayner would have world-wide recognition for this great discovery.

The moral is: Do not hide your light under a bushel, but report your cases and get the credit you deserve.

LEONARD PEARSON.

---

A Virginia firm of breeders have adopted a horn-fly tray, consisting of a dark room through which the cows are driven, and in passing through the flies are brushed off by hanging boughs of trees, etc., and closed up in the trap for a couple of days, when they die.

